# Extradural anaesthesia-analgesia in dogs undergoing cholecystectomy: A single centre retrospective study

**DOI:** 10.3389/fvets.2022.966183

**Published:** 2022-09-09

**Authors:** Beatrice Sambugaro, Chiara De Gennaro, Rachel D. Hattersley, Enzo Vettorato

**Affiliations:** Dick White Referrals, Linnaeus Veterinary Limited, Cambridgeshire, United Kingdom

**Keywords:** dog, extradural anaesthesia, extradural analgesia, extradural catheter, cholecystectomy

## Abstract

**Objectives:**

To assess the effects of extradural anaesthesia-analgesia (EAA) in dogs undergoing cholecystectomy.

**Materials and methods:**

Medical records of dogs undergoing cholecystectomy between 2011 and 2019 were retrieved and allocated to two groups depending if analgesia was provided systemically (group SA) or extradurally (EAA). Preoperative data, intraoperative antinociceptive medications, postoperative analgesia, perioperative complications, and food intake were compared.

**Results:**

Overall 41 medical records were included in the study: 19 and 22 dogs were allocated to groups SA and EAA, respectively. In group EAA, an extradural catheter was placed preoperatively in 8 dogs; in the remaining, it was placed postoperatively but an extradural injection was performed preoperatively. The extradural catheter tip was between the 4th lumbar and the 10th thoracic vertebrae. Intraoperatively, nociception was more likely to occur in group SA [OR 55.42 (2.97–1,035.06)]. During the first 24 and 48 h postoperatively, more dogs in group SA required methadone [OR 24 (2.81–268.4) and OR 11.56 (2.37–45.06), respectively] and additional analgesic drugs [OR 25 (3.47–281.9) and OR 35.29 (1.86–668.2), respectively] compared to group EAA. Voluntary postoperative food intake was also significantly higher in group EAA.

**Clinical significance:**

Compared to systemic analgesia, the use of extradural anaesthesia-analgesia reduced perioperative analgesic requirement and promoted postoperative food intake in dogs undergoing cholecystectomy.

## Introduction

Cholecystectomy is commonly performed in dogs affected by extrahepatic biliary tract obstruction (EHBO), gallbladder mucocoele or gallbladder rupture. In dogs, the mortality rate after cholecystectomy ranges between 2 and 40% (1–7). Extradural anaesthesia-analgesia (EAA) has been commonly used in dogs to decrease the requirement for inhalational anaesthetic agents and intraoperative opioids, to reduce the stress response and also postoperative opioid consumption (8–12).

The perioperative pain management of dogs undergoing cholecystectomy can be challenging, especially in presence of secondary pancreatitis and bile peritonitis. In our institution, the preoperative or the postoperative placement of an extradural catheter in dogs undergoing cholecystectomy has become more frequent during the last 5 years. It is our clinical impression that the use of EAA has improved the overall perioperative management of these dogs with minimal complications. For this reason, we designed this retrospective observational study in dogs undergoing cholecystectomy, hypothesising that, compared to systemic analgesia: (1) EAA would reduce the number of dogs requiring intraoperative interventions to control nociception (main outcome); (2) the postoperative use of an extradural catheter would reduce the need for opioid administration and additional analgesic drugs during the first 48 postoperative hours; (3) the use of an extradural catheter would increase the likelihood and amount of voluntary food intake during the first 48 postoperative hours.

## Materials and methods

### Study design and inclusion criteria

This was a single-centre, retrospective, observational, cross-sectional study. No ethical approval was pursued but written owner consent to use medical records for research purposes was obtained at the time of the dog's admission to our institution.

The medical records of dogs undergoing cholecystectomy at Dick White Referrals (UK) between 2011 and 2019 were identified by searching the theatre electronic database, and the practise management software, for the following keywords: cholecystectomy, EHBO, gallbladder mucocele, gallbladder rupture, epidural, extradural and dog. Retrieved medical records were excluded if (1) only a single preoperative or postoperative extradural injection was performed; (2) a preoperative extradural injection was performed using an opioid or a local anaesthetic only (LA); (3) other regional anaesthetic techniques were implemented; (4) a cholecystectomy was performed for reasons other than primary gallbladder disease; (5) euthanasia was performed intraoperatively or (6) the medical records were incomplete.

Dogs included in the study were assigned to one of the two following groups: group SA, where systemic analgesia was used for the entire perioperative period, and group EAA, where an extradural catheter was placed preoperatively and used for the entire perioperative period, or an extradural injection was administered preoperatively and an extradural catheter was placed at the end of the surgery.

Extradural injections and extradural catheters placement (Perifix® ONE; B Braun, Germany) were performed with the dog in sternal recumbency and with the pelvic limbs extended cranially. A Tuohy needle of adequate length was used (Perican® B Braun, Germany) and the extradural space was identified using the hanging drop and/or loss of resistance technique (13). The entrance point of the Tuohy needle, the targeted level of extradural catheter tip placement, opioid and LA dose, volume and concentration administered were at the discretion of the anaesthetist in charge of the case. Epidurography was not performed to confirm the exact position of the needle and the tip of the catheter.

### Data collection

Retrieved preoperative, intraoperative and postoperative information (Tables 1–5) were logged in an electronic spreadsheet (Microsoft Excel, Version 16.54). According to our internal laboratory reference ranges, preoperative anaemia was defined as haematocrit (Hct) < 37 L/L; leucocytosis as white blood cell count > 15 x 10^9^/L; hypoproteinaemia and hypoalbuminaemia as total protein and albumin < 54 and 25 g/L, respectively; elevated alanine aminotransferase (ALT) as ALT > 88 IU/L; hyperbilirubinaemia as bilirubin > 16 μmol/L; increased coagulation times as prothrombin and activated partial thromboplastin times > 12 and 25 s, respectively.

**Table 1:** Preoperative information recorded from a cohort of dogs undergoing cholecystectomy and in which systemic analgesia (SA) or extradural anaesthesia-analgesia (EAA) were used.

**Category**	**Information**	**SA** **(*n* = 19)**	**EAA** **(*n* = 22)**	***p*-value**	**OR (95% CI)**
Demographic data	Breed (n of dogs)	3 Miniature Schnauzer, 2 Border Terrier, 2 King Charles Spaniel, 2 cross breed, 2 Whippet, 2 Labrador, 6 others	11 Border Terrier, 2 Bichon Frise, 9 others		
	Sex (n of dogs)	12M (9MN)	8M (6MN)	0.12	
		7 F (7FS)	14F (13FS)		
	Age (months)	107 ± 43	126 ± 30	0.11	
	Body weight (kg)	13.5 (9.1–25)	9.5 (7.8–10.6)	0.04	
Clinical signs type, duration	Jaundice (n of dogs)	4 Y−15 N	7 Y−15 N	0.50	
& antiemetic administration	Lethargy/ hyporexia–anorexia/ weight loss (n of dogs)	12 Y−7 N	17 Y−6 N	0.52	
	Abdominal pain (n of dogs)	6 Y−13 N	4 Y−18 N	0.47	
	Vomiting–nausea (n of dogs)	9 Y−10 N	18 Y−4 N	0.026	5 (1.24–16.97)^*^
	Pancreatitis (n of dogs)	6 Y−13 N	3 Y−19 N	0.26	
	Duration of clinical signs (days):	10 (4–60)	7 (4–60)	0.74	
	Antiemetic / antiacids / prokinetics in dogs with vomiting (n of dogs)	5 Y−4 N	11 Y−7 N	>0.99	
	Types of Antiemetic / antiacid / prokinetic (n of dogs)	2 maropitant, 1 maropitant + ranitidine, 1 maropitant + metoclopramide 1 ranitidine	10 maropitant, 1 metoclopramide		
Preoperative blood work-up	Anaemia (n of dogs)	7 Y−12 N	9 Y−13 N	>0.99	
	Leucocytosis (n of dogs)	11 Y−8 N	10 Y−12 N	0.54	
	Hypoproteinaemia (n of dogs)	4 Y−15 N	5 Y−17 N	>0.99	
	Hypoalbuminaemia (n of dogs)	6 Y−13 N	8 Y−14 N	>0.99	
	Elevated ALT (n of dogs)	14 Y−5 N	20 Y−2 N	0.22	
	Hyperbilirubinaemia (n of dogs)	6 Y−13 N	12 Y−10 N	0.21	
	Increased coagulation times (n of dogs)	1 Y−18 N	1 Y−21 N	>0.99	
ASA status:		3 (3 - 3)	3 (3 - 3)	0.93	
Type of surgery	Elective (n of dogs)	13	8	0.06	3.79 (1.11–12.43)
	Emergency (n of dogs)	6	14		
Reason for surgery	Gallbladder mucocele (n of dogs)	12 Y−7 N	15 Y−7 N	>0.99	
	Cholangitis / cholangiohepatitis (n of dogs)	1 Y−18 N	2 Y−20 N	>0.99	
	Cholelithiasis (n of dogs)	1 Y−18 N	2 Y−20 N	0.61	
	Gallbladder rupture (n of dogs)	5 Y−14 N	3 Y−19 N	0.44	

Results are reported as number (n) of dogs, mean ± standard deviation, median (95% confidence intervals - CI), odd ratio (OR) or reciprocal OR with 95% CI.

n, number; M, male; MN, male neutered; F, female; FS, female spayed; Y, yes; N, no; ASA, American Society of Anesthesiologists physical status classification. Anaemia defined as haematocrit <37 L/L; leucocytosis as white blood cell count > 15 x 10^9^/L; hypoproteinaemia as total protein <54 g/L, hypoalbuminaemia as albumin <25 g/L; elevated alanine aminotransferase (ALT) as ALT > 88 IU/L; hyperbilirubinaemia as bilirubin > 16 μmol/L; increased coagulation times as prothrombin time > 12 s and activated partial thromboplastin time > 25 s.

* = reciprocal OR.

During surgery, the mean end-expiratory fraction of the inhalant anaesthetic agent administered to maintain anaesthesia was calculated by averaging the readings recorded at 5 min intervals (Wato EX-65 Mindray, UK). Multiples of minimum alveolar concentration (MAC) were then calculated considering isoflurane and sevoflurane MAC as 1.28 and 2.36%, respectively (14, 15).

Intraoperative nociception was defined as an increase of the analgesic infusion rate and/or the administration of another analgesic drug to correct an increase in heart rate or arterial blood pressure > 20% values compared to the previous recorded measurement (16). In the SA group, the baseline antinociceptive treatment was the one provided by the analgesic drugs administered before the beginning of the surgery. Further infusions, an increase in the infusion rate or the administration of a bolus of another analgesic drug to control nociception were defined as additional analgesic administration. In the EAA group, the baseline antinociceptive treatment was the one provided by the preoperative extradural injection; any further systemic drug administered to correct nociception was defined as additional analgesic administration. The number of dogs that required an adaptation of the baseline antinociceptive treatment, based on intraoperative nociception, was identified. Arterial blood pressure was measured either with an oscillometric or an invasive technique (Mindray BeneView T5). The width of the arterial blood pressure cuff used was approximately 40% of the circumference of the antebrachium or metatarsus at the level where it was placed (17). For invasive arterial blood pressure monitoring, the dorsal pedal artery was catheterised. The blood pressure transducer was zeroed at atmospheric pressure and was placed at the level of the right atrium (18). Hypotension was defined as mean arterial pressure (MAP) < 60 mmHg or systolic arterial pressure (SAP) < 90 mmHg lasting for at least 10 min (19). The use of drugs with a predominant positive inotropic effect (i.e., dobutamine, dopamine and ephedrine) or vasoconstrictive effect (i.e., noradrenaline and phenylephrine) was evaluated. Moderate hypothermia was defined as an oesophageal temperature < 36.5°C at the end of anaesthesia (20).

Postoperative rescue analgesia (methadone 0.2 mg/kg IV; Comfortan®; Dechra, UK) was administered based on pain scores which were determined every 2 h using the short form of the Glasgow Composite Measure Pain Scale (CMPS-SF) (21). The administration of postoperative non-steroidal anti-inflammatory drugs (NSAIDs) was also recorded. Additional postoperative analgesia was defined as the IV administration of analgesic drugs in addition to methadone ± NSAIDs (e.g., constant rate infusion (CRI) of opioids, alpha_2_ agonists, ketamine, lidocaine, or a combination of the above) if the pain scores were greater than the recommended analgesic intervention level. The presence of perioperative pancreatitis was diagnosed based on clinical signs, ultrasound findings and/or an increase in canine pancreatic lipase. The postoperative administration of antiemetic, prokinetic and antiacid medications and placement of oesophagostomy or gastric feeding tubes were recorded. The oesophagostomy tubes were placed at the end of the surgery. Information regarding the placement of postoperative urinary catheters, the need for postoperative urinary interventions and the time to first urination was collected for both groups. The latter was not considered in dogs that needed postoperative urinary interventions or had a urinary catheter placed at the end of surgery.

Time to the first voluntary food intake was recorded as hours from the end of the surgery, and the amount eaten during the first 24 and the 24–48 postoperative hours was calculated and expressed as a percentage of the resting energetic requirement (RER) (22).

### Statistical analysis

A sample size calculation was performed considering that 113 out of 119 (95%) dogs undergoing cholecystectomy received either fentanyl or alfentanil during surgery (7). To prove a 50% reduction in the number of dogs needing intraoperative opioid administration when EAA was used, with a power of 80% and an alpha error of 5%, at least 15 dogs per group were needed. Data distribution was assessed for normality using D'Agostino and Pearson test (GraphPad Prism version 8 for Mac; GraphPad Software Inc., California, United States). Continuous preoperative data were compared between groups using either a Student's *t*-test or a Mann-Whitney U test. The area under the curve (AUC) of the postoperative RER voluntarily eaten by the dog was calculated and compared between groups. Results are reported as mean ± standard deviation or median (95% Confidence Intervals–CI), depending on their distribution. Fisher's Exact test was used to compare ordinal data. Odds ratio (OR), or reciprocal OR, with 95% CI are reported when indicated. The Haldane-Anscombe correction was applied to the OR calculation if one of the values of the contingency table was 0 (23). A *p* < 0.05 was considered statistically significant.

## Results

**Figure 1:**
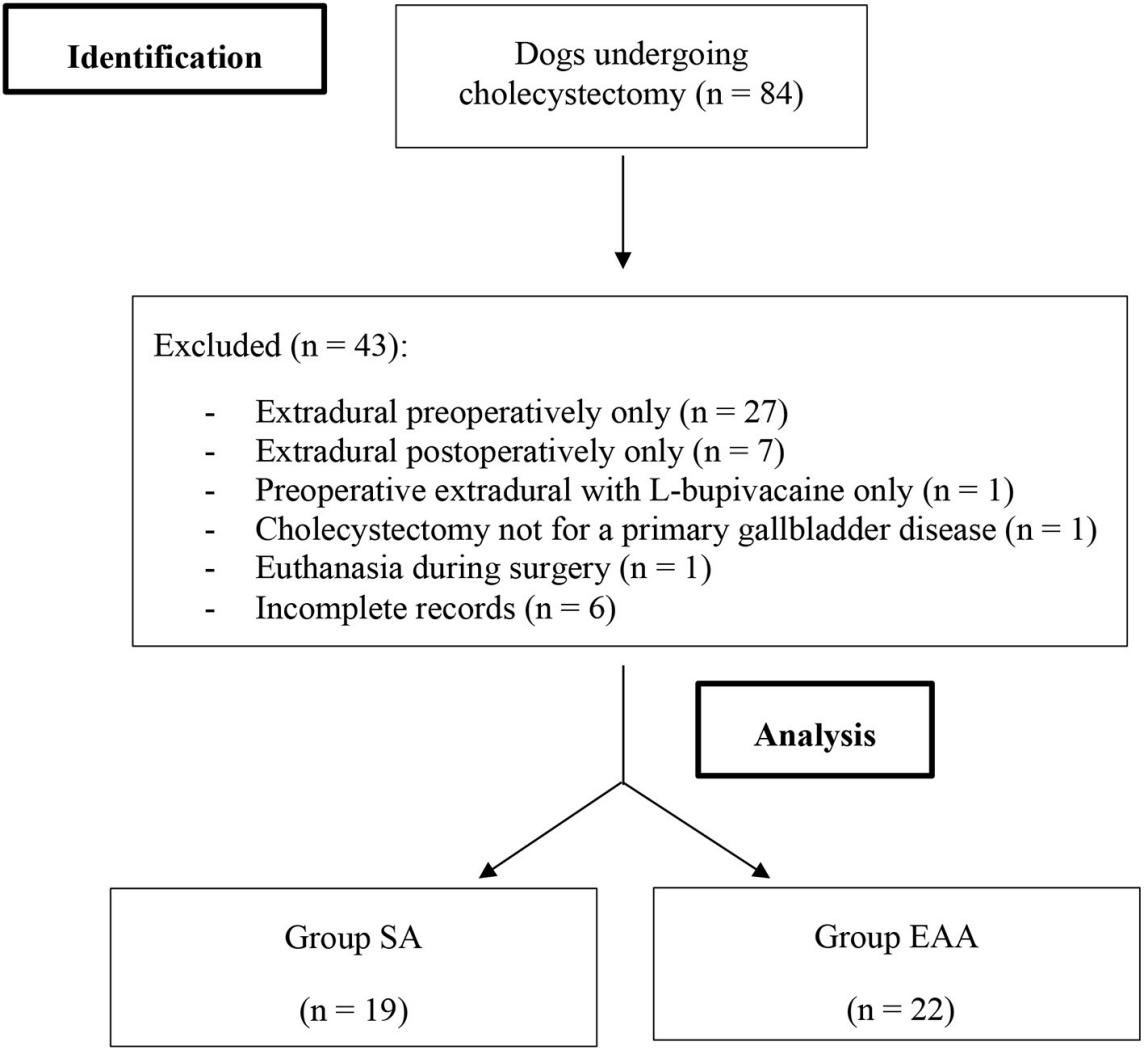
Consolidated Standards of Reporting Trials (CONSORT) flow diagram. n, number; SA, systemic analgesia; EAA, extradural anaesthesia-analgesia.

Of the 84 retrieved medical records, 41 met the inclusion criteria: 19 were assigned to group SA and 22 to the group EAA (Figure 1).

Signalment data were similar between groups (Table 1) but dogs in group SA were statically heavier in terms of body weight (*p* = 0.04). Clinical signs included jaundice, lethargy, hyporexia or anorexia, weight loss, abdominal pain, vomiting and nausea. The prevalence of preoperative pancreatitis was similar between groups. The prevalence of preoperative vomiting was statistically higher in group EAA. However, the overall number of dogs in which preoperative antiemetics, prokinetics and/or antacids had been administered was not different between the two groups (Table 1). The duration of clinical signs, haematological and biochemical blood abnormalities and the reason for performing cholecystectomy were also not significantly different between groups. In group SA, surgeries were more likely to be elective [OR 3.79 (1.11–12.43)] (Table 1).

The prevalence of dogs receiving only methadone (0.2 mg/kg IV) as pre-anaesthetic medication was higher in the group SA [p = 0.06; OR 4.04 (1.14–15.12)]. The type of induction and inhalational anaesthetic agents administered, and the MAC value were not different between groups (Table 2). While surgical time was not statistically different between groups, anaesthesia time was longer in the EAA group (Table 2).

**Table 2:** Intraoperative information recorded from a cohort of dogs undergoing cholecystectomy and in which systemic analgesia (SA) or extradural anaesthesia-analgesia (EAA) were used.

**Category**	**Information**	**SA** **(*n* = 19)**	**EAA** **(*n* = 22)**	***p* value**	**OR (95% CI)**
Premedication	Methadone (n of dogs)	14	9	0.06	4.04 (1.14–15.12)
	Methadone + alpha_2_ agonist (n of dogs)	5	13		
Induction agent	Propofol (n of dogs)	12	15	0.75	
	Alfaxalone (n of dogs)	7	7		
Inhalational anaesthetic agent	Isoflurane (n of dogs)	14	15	0.74	
	Sevoflurane (n of dogs)	5	7		
	MAC value	0.92 ± 0.1	0.87 ± 0.1	0.09	
Procedural time	Anaesthesia time (min)	115 ± 29	169 ± 43	<0.0001	
	Surgery time (min)	60 ± 21	75 ± 35	0.16	
Nociception	Additional analgesic administration (n of dogs)	19 Y−0 N	9 Y−13 N	<0.0001	55.42 (2.97–1035.06)
Arterial blood pressure	IBP–NIBP (n of dogs)	16 - 3	13 - 9	> 0.99	
monitoring	Hypotension (n of dogs)	6 Y−13 N	13 Y−9 N	0.54	
	Hypotension (n of events per dog)	1 (0−2)	1 (0 - 1)	0.37	
Treatment of hypotension	Antimuscarinic (n of dogs)	7 Y−12 N	4 Y−18 N	0.29	
	Positive inotrope (n of dogs)	3 Y−16 N	10 Y−12 N	0.05	4.44 (1.01–17.01)^*^
	Vasopressor (n of dogs)	3 Y−16 N	2 Y−20 N	0.65	
	Hartmann's rate (ml/kg/h)	5 (4–10)	5 (4–6)	0.23	
	Fluid boluses (n of dogs)	8 Y−11 N	17 Y−5 N	0.03	4.67 (1.27–18.3)^*^
	Total volume of fluid boluses (ml/kg)	9.2 (2–21.3)	9.5 (7.8–11.8)	0.58	
Body temperature	Oesophageal temperature at end of surgery (°C)	36.6 (36–37.5)	35.9 (35.4–36.6)	0.04	
	Oesophageal temperature <36.5°C (n of dogs)	8 Y−11 N	17 Y−5 N	0.03	4.67 (1.26–18.30)^*^

Results are reported as number (n) of dogs, mean ± standard deviation, median (95% confidence intervals - CI), odd ratio (OR) or reciprocal OR with 95% CI.

n, number; Y, yes; N, no; MAC, minimum alveolar concentration; IBP, invasive arterial blood pressure; NIBP, non-invasive arterial blood pressure.

* = reciprocal OR.

In group EAA, an extradural catheter was placed preoperatively in 8 out of 22 dogs. In the remaining 14, an extradural injection was performed preoperatively and an extradural catheter was placed at the end of surgery. L-bupivacaine (Chirocaine; Abbvie Ltd, UK), combined with either morphine (Morphine sulphate; Hameln, UK) or buprenorphine (Buprevet®; Virbac, UK), was used preoperatively. The median (95% CI) concentration of L-bupivacaine was 0.38 (0.19–0.48)%. Details regarding the execution of preoperative EAA (Tuohy needle entrance point, advancement of the extradural catheter, dose and volume of the drugs administered) and the use of intraoperative opioids in group EAA are reported in Table 3.

**Table 3:** Details regarding preoperative extradural and extradural catheter placement, drugs used and intraoperative opioid requirement in a cohort of dogs undergoing cholecystectomy.

**N of dogs**	**Tuohy needle**	**Catheter advancement**	**Drugs used**					
	**Entrance point**	**Catheter tip level**	**0.5% L-bupivacaine (mg/kg)**	**1% Morphine (mg/kg)**	**0.03% Buprenorphine (μg/kg)**	**Total volume (ml/kg)**	**L-bupivacaine final concentration (%)**	**Intraoperative opioid administered**
1	T13-L1	T10-T11	0.59	0.12		0.12	0.48	Fentanyl boluses
1	L2-L3	T13-L1	0.81		9.8	0.20	0.42	No
1	L6-L7	L2-L3	1.62	0.13		0.34	0.47	No
5	L7-S1	L1-L2	0.57		5.7	0.14	0.4	Alfentanil CRI
		L1-L2	1.35		8.1	0.54	0.25	No
		L2-L3	1.28	0.09		0.27	0.48	No
		L3-L4	0.64	0.09		0.19	0.33	No
		L3-L4	0.99	0.1		0.30	0.33	No
1	L1-L2	N/A	0.99	0.16		0.21	0.46	Alfentanil CRI
2	L6-L7	N/A	1.26 1.29	0.12 0.19		0.38 0.32	0.33 0.36	No
1	L6-L7	N/A	1.48		9.9	0.42	0.35	No
5	L7-S1	N/A	1.27 (0.67–2.1)^*^	0.1 (0.1–0.17)^*^		0.32 (0.18–0.47)^*^	0.44 (0.33–0.48)^*^	Fentanyl boluses in three dogs
5	L7-S1	N/A	1.98 (1.6–2.1)^*^		9.5 (9.4–10.3)^*^	0.64 (0.4–1.1)^*^	0.45 (0.19–0.46)^*^	Fentanyl boluses in three dogs

* indicates median (95%CI).

n, number; T, thoracic; L, lumbar; S, sacral; CRI, constant rate infusion; N/A, not applicable: the drug was injected at the entrance point and no extradural catheter was placed preoperatively.

In group SA, an infusion of an analgesic was started in 16 dogs before the beginning of the surgery. In particular, a fentanyl CRI (5–10 μg/kg/h; Fentadon®; Dechra, UK) was administered to one dog; a remifentanil CRI (0.05–0.3 μg/kg/min; Remifentanil; Wockhardt, UK) to nine dogs; a remifentanil CRI (0.05–0.3 μg/kg/min) and ketamine boluses (0.5 mg/kg; Anesketin; Dechra, UK) to one dog; an alfentanil CRI (0.5–1 μg/kg/min; Rapifen®; Piramal Critical Care, UK) to three dogs; lidocaine CRI (30–50 μg/kg/min; Lidocaine Hydrochloride; Hameln, UK) and fentanyl boluses (2 μg/kg) to one dog; lidocaine CRI (30–50 μg/kg/min) and fentanyl CRI (5–10 μg/kg/h) to one dog. In all of them, the infusion rate was increased or another analgesic drug was added to control nociception. Three dogs did not have an infusion at the beginning of the surgery but required intraoperative fentanyl boluses (1 μg/kg) to control nociception. In group EAA, 9 out of 22 dogs required the administration of additional analgesia to control nociception. In particular, fentanyl boluses were administered to seven dogs, and an alfentanil continuous infusion was started in two dogs (Table 3). Overall, the prevalence of dogs requiring an increase in the analgesic infusion rate and/or the administration of another analgesic drug to control nociception was statistically higher [*p* < 0.0001; OR 55.42 (2.97–1035.06)] in group SA (Table 2).

Arterial blood pressure data is summarised in Table 2. Intraoperatively, the number of dogs in which hypotension was recorded and the number of hypotensive events observed per dog were not different between groups (Table 2). Of the hypotensive dogs, five out of nine dogs [*p* = 0.06; OR 11.25 (1.16–144)] and seven out of 13 dogs [*p* = 0.38; reciprocal OR 3 (0.48–17.72)] underwent emergency cholecystectomy, in group SA and EAA respectively. The use of antimuscarinics and vasopressors was not different between groups (Table 2). However, a higher proportion of dogs in group EAA needed positive inotropes [*p* = 0.05; reciprocal OR 4.44 (1.01–17.010)] and fluid boluses [*p* = 0.03; reciprocal OR 4.67 (1.27–18.30)] intraoperatively. All the treatments were administered according to the anaesthetist's clinical judgment. The total volume of fluid administered intraoperatively was similar between groups (Table 2). At the end of the surgery, the number of dogs with moderate hypothermia was statistically higher in group EAA [*p* = 0.03; reciprocal OR 4.67 (1.26–18.30)].

In group EAA, one dog was euthanised 13 h after surgery due to a haemoabdomen and was therefore not included in the postoperative analysis. Furthermore, during the initial 24–48 h postoperatively, two further dogs in the group EAA were excluded from the analysis due to dislodgement of the extradural catheter and euthanasia due to the development of septic peritonitis, respectively. Extradural medications were administered postoperatively at fixed intervals (every 4–6 h) or based on pain scores depending on the anaesthetist's decision. If pain scores were still above 5/20 or 6/24 2 h after extradural administrations, methadone (0.2 mg/kg) was administered IV and the volume and/or concentration of the extradural LA was adjusted for the next administration. During the first 24 h postoperatively, extradural medications were administered every 4–6 h in 16 out of 21 dogs. In the remaining five dogs, the administration was based on pain scores. The number of postoperative extradural injections, the volume and the concentration of the solution administered in group EAA during the first 24 and between 24 and 48 h postoperatively are summarised in Table 4. The median (95% CI) number of administrations, volume and percentage of the L-bupivacaine solution administered were: 4 (4–5), 0.2 (0.15–0.26) ml/kg, 0.15 (0.13–0.2)%, respectively.

**Table 4:** Details regarding extradural catheter placement, number of postoperative administrations, volume and concentration of the L-bupivacaine used in a cohort of dogs undergoing cholecystectomy.

	**Tuohy needle**	**Catheter advancement**	**First 24 PO h**			**24-48 PO h**		
			**L-Bupivacaine administration**			**L-Bupivacaine administration**		
**N of dogs**	**Entrance point**	**Tip of the needle level**	**Administrations (n)**	**Volume (ml/kg/dose)**	**% solution**	**Administrations (n)**	**Volume (ml/kg/dose)**	**% solution**
1	T13-L1	T10-T11	4	0.12	0.17	2	0.12	0.17
1	L2-L3	T13-L1	5	0.32	0.25	2	0.32	0.17
1	L6-L7	L2-L3	2	0.32	0.14	0		
3	L6-L7	T13-L1	4	0.2	0.125	1	0.2	0.125
			5	0.13	0.125	5	0.18	0.125
			2	0.25	0.25	1	0.25	0.25
10	L7-S1	T13-L1	4	0.2	0.125	0		
			5	0.24	0.13	0		
			4	0.21	0.125	0		
			4	0.31	0.15	4	0.31	0.15
			4	0.13	0.2	3	0.13	0.2
			4	0.16	0.25	N/A		
			1	0.1	0.125	N/A		
			5	0.27	0.15	5	0.27	0.15
			2	0.21	0.25	0		
			5	0.2	0.15	4	0.25	0.14
2	L7-S1	L1-L2	4	0.14	0.13	0		
			6	0.54	0.25	4	0.54	0.25
1	L7-S1	L2-L3	5	0.19	0.17	6	0.19	0.17
2	L7-S1	L3-L4	3	0.15	0.17	1	0.15	0.17
			4	0.26	0.2	4	0.26	0.2

PO, postoperative; n, number; T, thoracic; L, lumbar; S, sacral; h, hours; N/A, not available as the dog was excluded from analysis because it was either euthanised or the extradural catheter was removed.

During the following 24–48 h postoperatively, all extradural injections were administered according to pain scores: 6 out of the 19 dogs did not require any extradural medication. Of these, two dogs were receiving a NSAID and one paracetamol (10 mg/kg three times a day *per os*; Paracetamol; Bova, UK). The median (95% CI) number of administrations, volume and percentage of the L-bupivacaine solution administered during the 24–48 h postoperatively were: 2 (0–4), 0.25 (0.15–0.31) ml/kg, 0.17 (0.14–0.2)%, respectively.

During the first 24 and the following 24–48 h postoperatively, the number of dogs needing postoperative methadone, the number of methadone administrations, and the number of dogs needing additional postoperative analgesia were greater in group SA (Table 5). During the first 24 hours postoperatively, methadone was administered regardless every 4 h in 12 out of 19 dogs in group SA. In the remaining seven dogs, it was administered based on pain scores. In addition to methadone, during the first 24 postoperative hours, a lidocaine CRI (33 μg/kg/min) was administered to 6 dogs, a lidocaine CRI and ketamine CRI (0.3–0.4 mg/kg/h) were administered to 2 dogs, a lidocaine CRI and a fentanyl patch (3 μg/kg/h) were used in one dog and a dexmedetomidine CRI (0.5–1 μg/kg/h) was administered to one dog in group SA. In one dog, no methadone was administered but analgesia was provided with lidocaine CRI and a NSAID. This case was not considered for the additional CRI calculation for the first 24 postoperative hours. Between 24 and 48 h postoperatively, methadone was administered according to pain scores in all dogs in group SA: at least one dose of methadone was administered to 13 out of 19 dogs. In addition to methadone, a lidocaine CRI was administered to seven dogs, ketamine CRI to one dog and a fentanyl patch applied to one dog in group SA. In group EAA, 42.9 and 15.8% of dogs required at least a dose of methadone during the first and second 24 postoperative hours, respectively. Only one dog required an additional ketamine CRI which was started at 17 h postoperatively; this dog was subsequently euthanised due to septic peritonitis. In group EAA, two dogs developed pelvic limb weakness after extradural administration of 0.25% L-bupivacaine. This problem was resolved by reducing the concentration to 0.2%.

**Table 5:** Postoperative information recorded from a cohort of dogs undergoing cholecystectomy and in which systemic analgesia (SA) or extradural anaesthesia-analgesia (EAA) were used.

**Information**		**SA** **(n of dogs = 19)**	**EAA** **(n of dogs = 19–21)^§^**	***p* value**	**OR (95%CI)**
PO NSAIDs	Administration (n of dogs)	9 Y−10 N	11 Y−10 N	>0.99	
	Robenacoxib (n)	5	9	0.33	
	Meloxicam (n)	2	2	>0.99	
	Carprofen (n)	2	0	0.22	
PO opioid requirement first 24 h	Methadone (n of dogs)	18 Y−1 N	9 Y−12 N	0.0006	24 (2.81–268.4)
	Methadone administrations (n)	5 (2–6)	0 (0–1)	<0.0001	
	Additional CRI (n of dogs)	10 Y−8 N	1 Y−20 N	0.0008	25 (3.47–281.9)
PO opioid requirement 24–48 h	Methadone (n of dogs)	13 Y−6 N	3 Y−16 N	0.0025	11.56 (2.37–45.06)
	Methadone administrations (n)	3 (0–5)	0 (0–0)	0.03	
	Additional CRI (n of dogs)	9 Y−10 N	0 Y −19 N	0.001	35.29 (1.86–668.2)
PO urination	Urinary catheter placement at end of surgery (n of dogs)	5 Y −14 N	6 Y −15 N	>0.99	
	PO urinary interventions (n of dogs)	0 Y −14 N	4 Y−11 N	0.10	
	Time to first urination (h)	7 (3–13)	7 (3–19)	0.78	
Pancreatitis^$^	Over 48 h (n of dogs)	7 Y−12 N	6 Y−15 N	0.74	
Vomiting / regurgitation	First 24 h (n of dogs)	3 Y−16 N	3 Y−18 N	>0.99	
	24-48 h (n of dogs)	2 Y−17 N	4 Y −15 N	0.66	
	Antiemetic / prokinetic / anti-acids administration (n of dogs)	10 Y−9 N	21 Y−0 N	0.0003	38.9 (2.06–734.44)^*^
	Maropitant (n of dogs)	7 Y−12 N	15Y−6 N	0.05	4.29 (1.20–15.01)^*^
PO feeding	Oesophagostomy or gastric feeding tube placement (n of dogs)	7 Y−12 N	17 Y−4 N	0.009	7.28 (1.78–24.93)^*^
	Duration of tube-feeding (days)	3 (1–9)	3 (1–5)	0.40	
	Time to first oral intake (h)	24 (10–40)	21 (12–42)	0.97	
	Voluntary food intake during first 24 h (n of dogs)	10 Y−9 N	11 Y −10 N	>0.99	
	AUC of RER eaten voluntary first 24 h	241 ± 187	428 ± 249	0.01	
	AUC of RER eaten voluntary second 24 h	499 ± 257	818 ± 300	0.001	
Overall outcome	Survival rate (n of dogs)	18 Y−1 N	19 Y−3 N	0.61	
	Hospitalisation time (days)	6 (4–8)	4 (4–6)	0.22	

Results are reported as number (n) of dogs, mean ± standard deviation, median (95% confidence intervals–CI), odd ratio (OR) or reciprocal OR with 95% CI.

PO, postoperative; n, number; NSAIDs, non-steroidal anti-inflammatory drugs; Y, yes; N, no; h, hours; AUC, area under the curve; RER, Resting Energy Requirements.

$ Pancreatitis refers to the overall prevalence: preoperative + postoperative.

§ One dog was excluded from the analysis during the first 24 postoperative hours because it developed haemoabomen and was euthanised; two dogs were excluded from the analysis during the 24–48 h because: 1) the extradural catheter was dislodged and was removed; 2) the dog was euthanised because of septic peritonitis.

* reciprocal OR.

Postoperative NSAIDs, paracetamol and gabapentin (10 mg/kg three times a day *per os*; Gabapentin; Milpharm, UK) administrations were at discretion of the clinician in charge of the case and were similar between groups (Table 5). Overall, paracetamol was administered to one and two dogs in group SA and EAA, respectively and gabapentin was administered to one dog in both SA and EAA groups.

No difference was identified in the number of dogs in which an indwelling urinary catheter was placed at the end of surgery between the two groups (Table 5). In group EAA, it was necessary to place an indwelling urinary catheter during the first 10 h postoperatively in an additional four dogs because the bladder was big and firm on palpation (three dogs) or because of constant dribbling of urine (one dog). The time to first urination was not different between groups (Table 5). The last extradural injection was administered at 32 (19–48) hours after surgery. Extradural catheters were removed 3 (2–4) days after placement.

Postoperative pancreatitis was diagnosed in one and three dogs in group SA and EAA, respectively. While the overall prevalence of pancreatitis, postoperative vomiting and/or regurgitation was not different between groups, the postoperative administration of antiemetic, prokinetic and antiacids medications, which was at discretion of the surgeon in charge of the case, was greater in group EAA [*p* = 0.0003; reciprocal OR 38.9 (2.06–734.44)]. In particular, during the first 24 h postoperatively, the administration of maropitant (Cerenia; Zoetis, UK) was more prevalent in the EAA group [p = 0.05; reciprocal OR 4.27 (1.20–15.010]. Similarly, the placement of gastric or oesophagostomy tubes, which was at discretion of the surgeon in charge of the case, was significantly higher in group EAA [p = 0.009; reciprocal OR 7.28 (1.78–24.93)]. The duration of use of feeding tubes, the number of dogs that ate voluntarily within the first 24 postoperative hours and the time to first spontaneous oral intake were not different between groups (Table 5). However, the AUC of the RER eaten voluntarily during the first 24 and the subsequent 24–48 h postoperatively was greater (p = 0.01 and 0.001, respectively) in group EAA (Table 5).

The overall survival to discharge was 90.2% and no difference was found between the two groups (Table 5). In group SA, one dog was euthanised 3 days after surgery following a diagnosis of high-grade lymphoma. In group EAA, one dog was euthanised 13 h after surgery because of haemoabdomen, one dog was euthanised 27 h after surgery because of septic peritonitis and one dog was euthanised 5 days after surgery because of necrotic common bile duct and septic peritonitis. Overall hospitalisation time was not different between groups (Table 5).

## Discussion

To the author's knowledge, this is the first study reporting the use of EAA, and evaluating its efficacy, in dogs undergoing cholecystectomy. According to our results, the perioperative use of EAA reduced the perioperative opioids consumption, the postoperative need of additional IV analgesic drugs, and increased the amount of voluntary food intake, supporting our hypothesis.

Providing effective analgesia in dogs undergoing cholecystectomy can be very challenging and the use of regional anaesthetic techniques can be complicated considering the complexity of the gallbladder innervation. The gallbladder possesses a dual innervation, sympathetic and parasympathetic, derived from the coeliac plexus. Fibres from the right phrenic nerve also appear to reach the gallbladder through the communication between the phrenic plexus and the coeliac plexus in the hepatic-plexus (24). Experimental studies in guinea pigs and cats showed that the gallbladder wall is innervated by sensory neurons that are located in the nodose ganglia and in the dorsal root ganglia between C5 and L3 (25), with the majority located between T2–L3 in cats (26, 27). Spinal afferent fibres travel mainly *via* the greater and lesser splanchnic nerves and through the right phrenic nerve (C5-C7) (25). Therefore, in order to provide a complete block, the extradural LA should spread above the thoraco-lumbar junction, ideally up to the last cervical vertebrae.

All dogs in group SA required an adaptation of the baseline antinociceptive treatment, by the administration of additional analgesics. On the contrary, in 59.1% of our cases, EAA was effective in blocking nociception, and no further interventions were required. However, intraoperative opioid administration was required in nine dogs. In six of these dogs, the EAA was injected lumbosacrally and an extradural catheter was not placed preoperatively. The extent of the sensory block produced by EAA is affected by the volume and the cranial spread of the solution administered (28–30). Therefore, in dogs undergoing cholecystectomy, a high volume of LA should be injected if the extradural administration is performed lumbosacrally. A more cranial extradural injection (i.e., thoraco-lumbar), or the placement of an extradural catheter, which tip could reach higher spinal segments, allows a reduction in the overall volume of LA, and therefore the risk of cardiovascular complications (31).

The volumes and concentrations of L-bupivacaine injected in this study were at the discretion of the anaesthetist in charge of the case. The final volume to be injected was generally calculated considering the point of injection or the position of the tip of the extradural catheter, and the percentage of the occipitococcygeal length to be blocked (32). Unfortunately, this specific information was not available in the medical records, and this is one of the limitations of this study. While it is possible that in some dogs the spread achieved was not sufficient to provide a complete block, other variables, such as age, weight, abdominal pressure, engorgement of extradural veins, amount of extradural fat, extradural pressure, dural surface area and experience of the operator could have played a role on the overall efficacy of EAA (33, 34). Nociception was recorded in two dogs in which an extradural catheter was placed preoperatively. Lateral placement of the catheter tip, presence of air bubbles and fibrous tissue formation have been reported as possible causes of uneven blockade in humans where extradural catheters were used (35–37). As epidurography was not performed, the exact location of the catheter tip was unknown, hence the reason for the block failure can only be speculated.

Preoperatively, all dogs included in the EAA group received L-bupivacaine combined with an opioid, either preservative-free morphine or buprenorphine. The addition of an opioid to the extradural LA administration produces a greater reduction of inhalational anaesthetic requirement during surgery, pain scores and postoperative rescue analgesia in dogs (8, 38, 39). Both morphine and buprenorphine can provide analgesia for up to 24 h when administered extradurally in dogs (8, 33, 40).

Previously, hypotension was reported in 54.6–74% of dogs undergoing cholecystectomy (7, 41). In our study, the overall prevalence of intraoperative hypotension was 53.7%: 47.4% and 59.1% in the SA and EAA groups, respectively. While this difference was not statistically significant, the increased administration of positive inotropes and fluid boluses recorded in the EAA group might indicate a clinically relevant difference between groups, even considering that among the inotropes, drugs with a potential vasoconstrictive effect were included. In dogs anaesthetised with inhalational anaesthetics, the sympathetic blockade caused by the extradural LA administration might have caused further vasodilation and hypotension (42, 43). On the contrary, the vasoconstriction caused by nociception could have masked the prevalence of hypotension in group SA. However, dog-related factors including pre-existing cardiac disease, hypovolaemia, dehydration and sepsis should also be considered. Dogs undergoing emergency surgery are more prone to intraoperative hypotension and could require more pharmacological interventions in order to maintain adequate arterial blood pressure (44). However, while the number of emergency surgeries was greater in the EAA group, hypotension was not more likely to occur. Instead, hypotension was 11.25 times more likely to occur during emergency cholecystectomies in the SA group. Therefore, a relationship between EAA and the prevalence of hypotension in dogs undergoing cholecystectomy could not be clearly demonstrated.

The vasodilation caused by extradural LA administration can also affect the redistribution of heat and the autoregulation of body temperature (45). The redistribution of body heat from the core compartment to the periphery is generally associated with an increased heat loss and can trigger hypothermia. During the first hour of extradural anaesthesia, vasodilation has been reported to contribute to 89% of core-to-periphery body heat redistribution (45). This could explain why, in our study, the temperature at the end of the surgery was significantly lower in the EAA group, compared to group SA, despite the use of active warming devices. The longer anaesthesia time recorded in group EAA, that could be explained by the time required to perform the extradural injections, the time to place the extradural catheters and the time needed to place the oesophagostomy tubes could have also contributed to the lower temperature recorded (20).

In our study, the postoperative administration of LA through the extradural catheter reduced the overall consumption of systemic analgesics. No opioids were required in 57.1% and 84.2% of dogs during the first 24 and 24–48 h postoperatively in group EAA. A single dog in the EAA group required a ketamine CRI which was presumed to be due to the development of septic peritonitis. This is in contrast to Hansen (46), who reported that only 27% of their case cohort, where an extradural catheter was used, did not receive additional systemic opioids. The difference between the two studies could be related to the different positions of the extradural catheter tip and the volume, concentration, frequency of LA administration and how pain was evaluated. In two experimental studies in beagle dogs, lidocaine was injected through an extradural catheter at the level of T7 (47) and T3 or T11 (48). In both studies, 0.2 ml/kg produced homogeneous sensory blockade of the thoracic region, while the lowest volume (0.05 ml/kg) resulted in unilateral, non-homogeneous blockade. In our study, the tip of the catheter was positioned approximately between T10 and L4 and the median volume of L-bupivacaine administered during the first and second 24 h was 0.2 ml/kg and 0.25 ml/kg, respectively. These volumes and catheter tip locations seemed appropriate to provide postoperative analgesia in most of the dogs that underwent cholecystectomy. After EAA administration, the duration and the degree of the block mainly depend on the drug used and the concentration of the solution (49). Modifying the LA concentration can produce a differential or selective block, with lower concentrations promoting a sensory over a motor blockade (50). In parturient women, L-bupivacaine was used at a lower concentration (0.125%) to provide analgesia without affecting the motor function (51). In our study, the median concentration of extradural L-bupivacaine was 0.38%, preoperatively, but it was decreased to 0.15–0.17%, postoperatively. The latter concentrations administered postoperatively seemed appropriate to provide analgesia, without compromising motor function in the majority of the cases.

In our study, one dog experienced dislodgment of the extradural catheter and two dogs developed pelvic limb weakness, which resolved by decreasing the LA concentration (from 0.25 to 0.2%). Catheter dislodgement (16%), inflammation (2.4%) or contamination (2.4%) of the catheter site have all been reported as potential complications associated with extradural catheter placement in dogs (52). Furthermore, Horner's syndrome, paraplegia, ataxia, depression, stupor, drowsiness, stridor and cough have also been reported following the extradural thoracic administration of lidocaine in dogs (47, 48). The lower complication rate reported in our study could be explained by the less extensive cranial spread, the lower concentration of the solution and the lower neurotoxicity of L-bupivacaine compared to lidocaine (53). Lateralisation of the catheter tip or incorrect catheter placement, could not be ruled out in our study as epidurography was not performed.

During abdominal surgery, the increased sympathetic tone secondary to the surgical stress response, excessive intravenous fluids administration which can cause bowel oedema, and the use of systemic opioids, may predispose to gastrointestinal ileus, constipation, nausea and vomiting (54, 55). By blocking sympathetic innervation and decreasing postoperative opioid consumption, extradural administration of a LA, with or without opioids, has been shown to accelerate the gastrointestinal transit but not to affect the incidence of vomiting in humans (56, 57). In our study, no difference was identified between groups regarding the prevalence of postoperative vomiting/regurgitation and/or pancreatitis despite the reduction in opioid consumption in the EAA group. While methadone does not generally cause vomiting in dogs, it could cause nausea and decrease gastric emptying affecting mainly the voluntary food intake (58, 59). In our study, the time to first voluntary food intake was similar between groups, whereas the amount of voluntary food intake was higher in the EAA group during both the first 24 and the subsequent 24–48 h postoperatively. As maropitant was more frequently administered to dogs in group EAA, and it has been shown to increase the postoperative food intake in a previous study (60), it is possible that its administration affected food intake between group SA and EAA.

In a recent multicentric study, 86.6% of dogs undergoing cholecystectomy survived to discharge (7). In that study, EAA was used in only 21% of dogs. According to our results, survival to discharge was 94.7% and 86.4% in groups SA and EAA, respectively. However, in group EAA, surgeries were 3.79 times more likely to be classified as emergency, and emergency surgeries are generally associated with higher mortality risk (61). Therefore, as used in this study, EAA does not negatively affect the overall outcome.

The main limitation of this study is the retrospective nature of the data leading to differences in protocols used. Anaesthesia protocols, the baseline antinociceptive treatment, the placement and use of the extradural catheter and intraoperative/postoperative analgesic choices were at discretion of the anaesthetist in charge of the case, and thus not standardised. Furthermore, the exact location of the tip of the catheter was not confirmed by epiduraography, therefore the calculation of the volume injected and the percentage of the spinal cord to be blocked was determined based on the presumptive location. Additionally, the anaesthetist in charge of the case and the observers scoring the postoperative pain were not blinded to the drugs administered. The decision on the use of pre and postoperative anti-emetic medications, the placement of a feeding tube and urinary catheter was also surgeon dependent. While all these limitations might have affected our results, we believe that this study represents a real clinical scenario and has clinical value. In particular, in this case cohort, the use of EAA reduces the number of dogs requiring intraoperative interventions to control nociception and the use of an extradural catheter reduces the requirement for postoperative opioids and additional IV analgesic drugs administration. Extradural anaesthesia-analgesia appears also to promote postoperative food intake with minimal complications. While prospective randomised studies are needed to confirm these results, the use of an extradural catheter appears to be beneficial in the perioperative management of dogs undergoing cholecystectomy.

## Data availability statement

The raw data supporting the conclusions of this article will be made available by the authors, without undue reservation.

## Ethics statement

Ethical review and approval was not required for the animal study because retrospective observational study. Written informed consent was obtained from the owners for the participation of their animals in this study.

## Author contributions

BS: data collection, data analysis, literature research, and manuscript preparation and writing. CD: study design and manuscript preparation, writing, and correction. RH: manuscript preparation, correction, and revision. EV: study design, statistical analysis and interpretation, and manuscript preparation, writing, and revision. All authors contributed to the article and approved the submitted version.

## Funding

This study received funding from Linnaeus Veterinary Limited. The funder was not involved in the study design, collection, analysis, interpretation of data, and the writing of this article or the decision to submit it for publication.

## Conflict of interest

Authors BS, CD, RH, and EV were employed by Linnaeus Veterinary Limited.

## Publisher's note

All claims expressed in this article are solely those of the authors and do not necessarily represent those of their affiliated organizations, or those of the publisher, the editors and the reviewers. Any product that may be evaluated in this article, or claim that may be made by its manufacturer, is not guaranteed or endorsed by the publisher.
